# Modified plant architecture integrated with liquid fertilizers improves fruit productivity and quality of tomato in North West Himalaya, India

**DOI:** 10.1038/s41598-021-98209-z

**Published:** 2021-09-20

**Authors:** Avinash Chandra Rathore, Harsh Mehta, J. Jayaprakash, Charan Singh, Anand Kumar Gupta, Pawan Kumar, Sadikul Islam, Saswat Kumar Kar, Sridhar Patra, Lekh Chand, Vijay Kumar Doharey, Prabhat Ranjan Ojasvi, Ram Swaroop Yadav, M. Madhu

**Affiliations:** 1 ICAR-Indian Institute of Soil and Water Conservation, 218 Kaulagarh Road, Dehradun, 248195 Uttarakhand India; 2 Krishi Vigyan Kendra, Jyolokot, Nainital, Uttarakhand India

**Keywords:** Biological techniques, Plant sciences, Environmental sciences

## Abstract

India produces around 19.0 million tonnes of tomatoes annually, which is insufficient to meet the ever-increasing demand. A big gap of tomato productivity (72.14 t ha^–1^) between India (24.66 t ha^–1^) and the USA (96.8 t ha^–1^) exist, which can be bridged by integrating trellis system of shoot training, shoot pruning, liquid fertilizers, farmyard manure, and mulching technologies. Therefore, the present experiment was conducted on tomato (cv. Himsona) during 2019–2020 at farmers' fields to improve tomato productivity and quality. There were five treatments laid in a randomized block design (RBD) with three replications; T_1_ [Farmer practice on the flatbed with RDF @ N_120_:P_60_:K_60_ + FYM @6.0 t ha^−1^ without mulch], T_2_ [T_1_ + Polythene mulch (50 microns)], T_3_ [Tomato plants grown on the raised bed with polythene mulch + FYM @ 8.0 t ha^−1^ + Single shoot trellis system + Side shoot pruning + Liquid Fertilizer (LF_1_—N_19_:P_19_:K_19_) @ 2.0 g l^–1^ for vegetative growth + Liquid Fertilizer (LF_2_—N_0_: P_52_: K_34_) @ 1.5 g l^–1^ for improving fruit quality], T_4_ [Tomato plants grown on the raised bed with polythene mulch + FYM @ 8.0 t ha^−1^ + Single shoot trellis system + Side shoot pruning + LF_1_ @ 4.0 g l^–1^ + LF_2_ @ 3.0 g l^–1^], and T_5_ [Tomato plants grown on the raised bed with polythene mulch + FYM @ 10.0 t ha^−1^ + Single shoot trellis system + Side shoot pruning + LF_1_ @ 6.0 g l^–1^ + LF_2_ @ 4.5 g l^–1^]. The results revealed that tomato plant grown on the raised beds with polythene mulch, shoot pruning, trellising, liquid fertilizers, and farmyard manure (i.e., T_5_) recorded higher shoot length, dry matter content, and tomato productivity by 20.75–141.21, 18.79–169.4, and 18.89–160.87% as compared to T_4_–T_1_ treatments, respectively. The T_5_ treatment also recorded the highest water productivity (28.39 kg m^–3^), improved fruit qualities, net return (10,751 USD ha^–1^), benefit–cost ratio (3.08), microbial population, and enzymatic activities as compared to other treatments. The ranking and hierarchical clustering of treatments confirmed the superiority of the T_5_ treatment over all other treatments.

## Introduction

**Figure 1:**
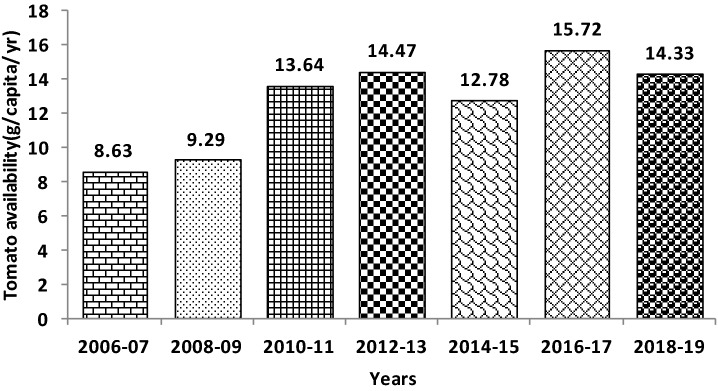
Tomato availability in India (Kg capita^−1^ year^−1^). *Source* FAOSTAT 2019 and NHB database 2018.

The tomato (*Solanum lycopersicum* L.), a member of the family Solanaceae is one of the world’s most consumed vegetable crops. It is one of the most important global vegetable crops because of its diversified uses, taste, colour, and high nutritive values. It is grown in tropical to temperate climatic zones, but tomatoes' actual yield in the tropics is generally low compared to the temperate regions. The global tomato production is 182.3 million tonnes from about 5.0 million ha area. China is the leading tomato producer (61.63 million tons from 1.03 million ha), followed by India (19.38 from 0.79). Based on tomato productivity (Table 1), the United States of America ranks first (96.8 t ha^–1^), followed by Turkey (68.86), China (59.25), Egypt (40.97), and India (24.66 t ha^–1^). However, India is the second-largest tomato producing country in the world after China and contributes 10.63% of global tomato production from 15.72% of the global tomato area^1^. Among the leading states, Andhra Pradesh recorded the highest tomato productivity (44.50), followed by Himachal Pradesh (42.88), Uttar Pradesh (39.62), and Karnataka (32.40). Tomato availability of India rose to 14.33 kg capita^–1^ year^–1^ (39.33 g capita^–1^ day^–1^) in 2018 from 8.63 kg capita^–1^ year^–1^ in 2006 with a rise of almost 66.0% (Fig. 1), which is also far below than tomato availability of 82.83 kg capita^–1^ year^–1^ (227.0 g capita^–1^ day^–1^) of Italy due to lower tomato productivity of our country^2^. Tomato productivity of India has been realized as high as 170.0 tha^–1^ in polyhouse condition^3^ which indicate that the potential of tomato productivity under protected cultivation (poly houses/net houses) can be harnessed outside the polyhouse/net house at farmers field, which will not only improve the tomato productivity but also increase national productivity (> 100 t ha^–1^) in open conditions^4^ by the plant architecture modification such as single shoot training (trellising), aside shoot pruning, mulching, and application of liquid fertilizers in India.

**Table 1:** Global area, production, and productivity of tomato in 2018.

Countries	Area (m ha)		Production (mt)		Productivity (t ha^−1^)		Global share (%)	
	2010	2018	2010	2018	2010	2018	2010	2018
China	0.871	1.040	41.88	61.63	48.08	59.25	28	33.81
India	0.865	0.786	16.83	19.38	19.46	24.66	11	10.63
Turkey	0.304	0.176	10.05	12.15	33.06	68.86	7	6.66
USA	0.159	0.137	12.90	12.61	81.13	96.80	9	5.92
Egypt	0.216	0.161	8.55	6.62	39.58	40.97	6	3.63
Total	2.42	2.33	90.21	111.30	37.35	47.87	61	61.65
Global	4.58	5.00	150.51	182.30	32.86	36.46	100	100

Source: FAOSTAT, 2019.

The major demerit of farmer practice of tomato cultivation (tomato plant without the support and pruning) is that tomato plant becomes more bushy, branchy, compact and restrict vertical growth (< 1.0 m) in tomato which interrupt proper ventilation/aeration inside the plant leads poor growth and attracts more pest due to higher humidity inside the plant. The single shoot trellis system of training aims to support plant growth in a desired shape/direction, which provides a better opportunity to harvest solar energy vertically to increase flowering and fruit yield in tomatoes. It also allows efficient air circulation, easy to practice cultural operations viz; weeding, hoeing, watering, harvesting, inspection of the field, prevent lodging, fruit rotting, liquid fertilizers application, etc^5,6^. This offers an opportunity to develop a training system with poles, wire, and threads to train the plants in a required shape into a trellis system of training, which will improve the duration of the flowering and fruiting total length of the main tomato shoot in addition to a stable support. In this practice, pruning parts of the plants like non–productive shoots, diseased leaves, and fruits are removed to regulate canopy and minimize photosynthates wastage. Tomato plants can be pruned severely without affecting the tomato yield^7,8^. Besides, the application of farmyard manure (FYM) also adds organic matter to the soil, which improves microbial population and enzymatic activities.

The current practice of applying solid fertilizers spoils soil health and decreases crop productivity in the long run compared to liquid fertilizer. Liquid fertilizer is a solution containing one or more nutrients essential to the plant, and its application can be arranged according to the plants' requirement as plants quickly absorb the nutrients and improve plant health, yields, fruit qualities (colour, carotenoid, vitamin C, etc.) and profitability. The nutrient use efficiency of nitrogen, phosphorus, and potash in India is 50, 10, and 40%, respectively, besides ill effects on soil health of major solid fertilizers (urea, diammonium phosphate, and muriate of potash). In other words, 50% of nitrogen, 90% phosphorus, and 60% potash is going out of the system as runoff or escaping into the atmosphere, causing other problems in water bodies^9,10^. It is an alternative to soil nutrient management and can be a better option for commercial use in horticulture as it provides nutrients as per the needs of crop^11,12^.

Therefore, this study aimed at applying plant architectural modification (pruning, trellising/training), and use of liquid fertilizers for open conditions at farmer’s fields to determine the impact on the performance of fruit yield and quality of tomato.

## Materials and methods

### Field location and descriptions

**Figure 2:**
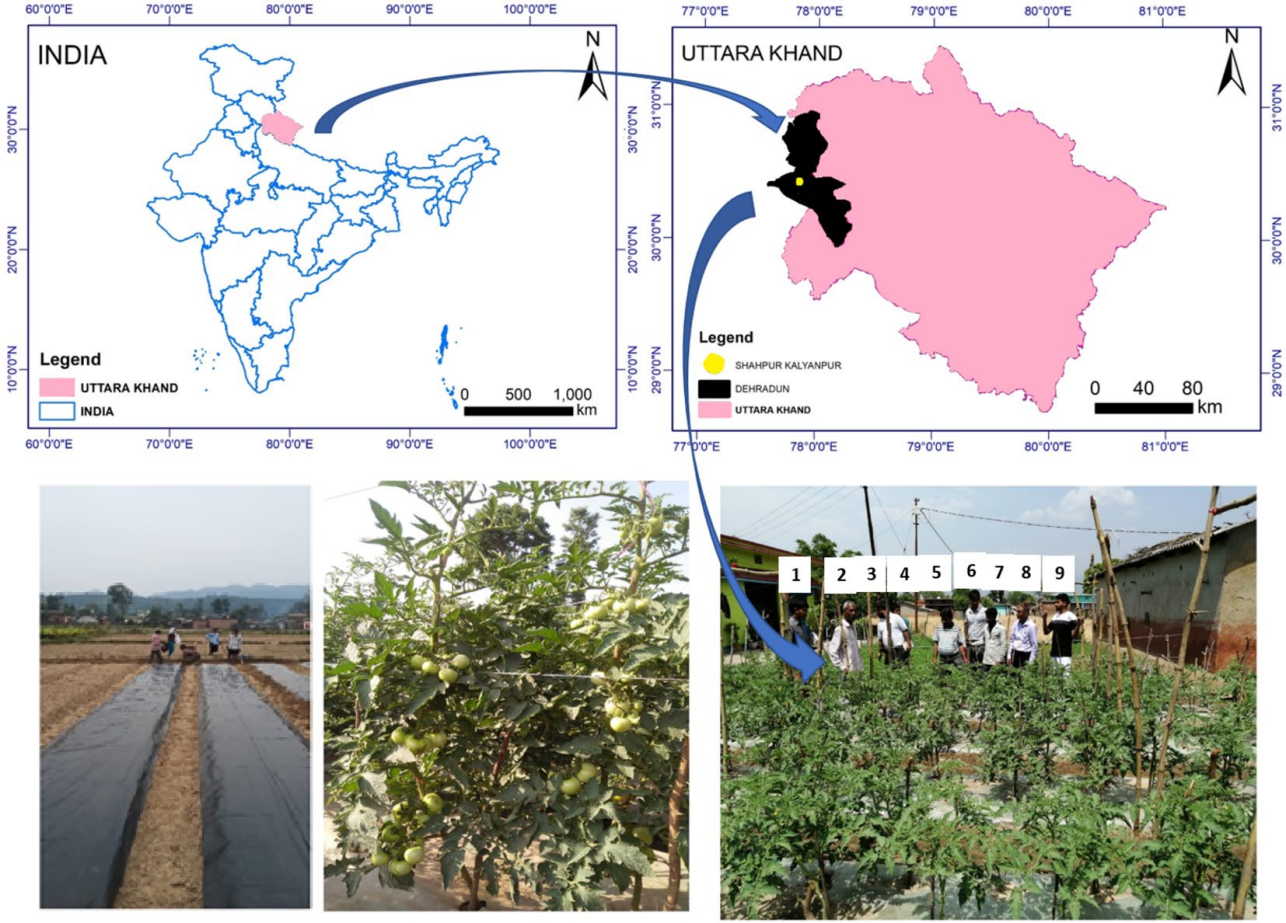
Location map of the experimental site. This map was prepared using Arc GIS 10.3. Humans appearing in the above figure are members of research team marked as 3, 5, 6, 8 and 9 as well as farmers (1, 2, 4 and 7). Informed consent for online publication of information and participation has been obtained from all the farmers**.**

The experiment was conducted on an indeterminate tomato cv. Himsona during March–July 2019–2020 in the tribal belt of Shahpur–Kalyanpur in Tehsil Vikasnagar of district Dehradun, Uttarakhand. This research involving tomato cv. Himsona complies with relevant institutional, national, and international guidelines and legislation and it does not involve collection of specific plant materials from the experimental area**.** This variety was released, identified and notified through All India Coordinated Research Project on Vegetable Crops (AICRP-VC) in 2009, and is in public domain in India. The experimental site is situated in the Indian Sub–Himalayan region at an altitude of 517 m above mean sea level between 30° 24′ 27″ N latitude and 77° 47′ 50″ E longitude (Fig. 2). The climate is humid subtropical, average annual rainfall of 1600 mm (approx. 80% during June–September) with a monsoonal rainfall pattern, mean monthly maximum and minimum temperature ranging from 19.0 to 37.6 °C in summer and 3.6–24.0 °C in the winter. Standard procedures were followed to determine the initial soil physical and chemical parameters replicated thrice for each treatment. The soil is classified as sandy loam texture. The soil pH ranged from 6.6–7.5, bulk density, 1.41–1.46 Mg m^–3^, soil organic carbon, 0.7–0.8%, total nitrogen, 242–265 kg ha^–1^, available phosphorus, 9.5–10.8 kg ha^–1^, and available potassium, 98.6–114.6 kg ha^–1^. Analysis of variance of these soil properties did not show any significant difference (p ≤ 0.05) among the samples collected from different treatment plots indicating a fairly homogeneous initial soil unit.

### Details of the experiment

**Figure 3:**
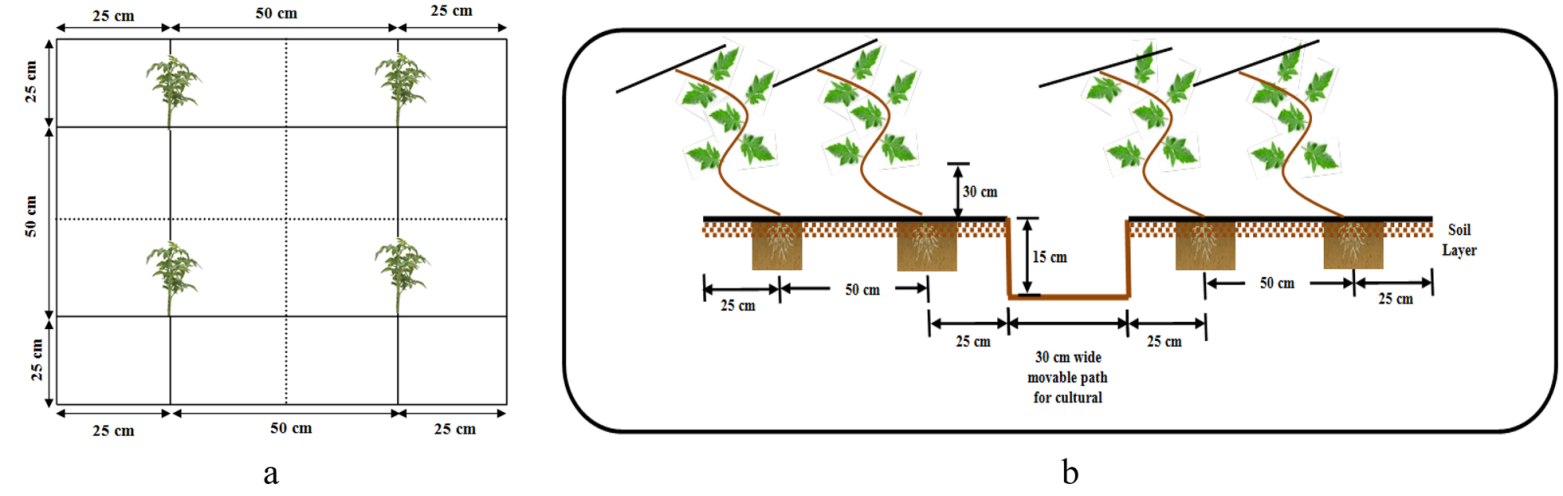
(**a**) Experimental layout of tomato field. (**b**) Planting design of tomato.

Seeds of indeterminate tomato (cv. Himsona) were sown in the first week of February during 2019 and 2020. About one-month-old seedlings were transplanted in the field in a paired rows system (65 cm × 45 cm) on 1.0-m-wide and 15 cm raised bed with a 30 cm channel between two beds to facilitate cultural operations such as weeding, training, pruning, pesticides spraying, harvesting, etc. The plant density maintained during both years was 34,188 plants ha^–1^. The black polythene mulch (50 microns) was laid on beds before tomato transplanting. The axillary shoots were removed after 25 days after transplanting, and after that, regular pruning of axil shoots was done at weekly intervals. The trellising of tomato seedlings (bamboo sticks/iron pole) was started after one month of tomato transplanting in the field. Plants were trained along the plastic thread tied to a galvanized iron wire stretched in a trellis system along the bed (Fig. 3). The tomato plants were irrigated manually using a flexible rubber pipe based on visual interpretation of plant symptoms and soil moisture conditions during March–June (2019–2020). In the initial phases of plant establishment (March 2019), each plant received approximately 200 ml per day and was increased to 800 ml per day towards the termination phase. The irrigation practice traditionally followed by local farmers was based on the visual interpretation of soil moisture and crop wilting stage. The water amount was determined using knowledge of the local farmers^13^. The irrigation was applied from day 1 (transplanting day) up to mid of June as per the need for different treatments. However, irrigation was withdrawn after monsoon arrival in June for both years.

Recommended doses of fertilizers (RDF) of nitrogen, phosphorus, and potash @ N_120_:P_60_:K_60_ kg ha^–1^ were applied as per the recommended procedure. N_60_:P_60_:K_60_ kg ha^–1^ was applied as basal dose, remaining N_60_ kg was applied in split doses (N_30_ and N_30_) of 20 and 60 days after transplanting in the field manually. Two formulations of liquid fertilizers (N_19_:P_19_:K_19_) and (N_0_:P_52_:K_34_) were applied in which (N_19_:P_19_:K_19_) was applied @ 2.0, 4.0, and 6.0 g l^–1^ at 15–20 days intervals for maintaining vegetative growth of tomato from transplanting in the field to final fruit harvesting time whereas second liquid fertilizer formulation N_0_:P_52_:K_34_ applied @ 1.5, 3.0 and 4.5 g l^–1^ after fruit initiation at 15–20 days intervals after flower initiation for yield and quality improvement of tomato fruits. The details of treatments studied on tomato plants are as follows:

**Table:** 

T_1_	Farmer practice on the flatbed with RDF @ N_120_:P_60_:K_60_ + FYM @6.0 t ha^-1^ without mulch
T_2_	T_1_ + Polythene mulch (50 micron)
T_3_	RBPM + FYM @ 8.0 t ha^−1^ + SSTS + SSP + LF_1_ @ 2.0 g l^–1^ + LF_2_ @ 1.5 g l^–1^
T_4_	RBPM + FYM @ 8.0 t ha^−1^ + SSTS + SSP + LF_1_ @ 4.0 g l^–1^ + LF_2_ @ 3.0 g l^–1^
T_5_	RBPM + FYM @ 10.0 t ha^−1^ + SSTS + SSP + LF_1_ @ 6.0 g l^–1^ + LF_2_ @ 4.5 g l^–1^

[RBPM (Tomato grown on raised bed with polythene mulch), RDF (Recommended dose of fertilizers), FYM (Farm Yard Manure) + SSTS (Single shoot trellis system), SSP (side shoot pruning) + LF_1_ (Liquid Fertilizer) supplied through (N_19_:P_19_:K_19_) for vegetative growth (15–20 days interval) during entire growing period + LF_2_ (Liquid Fertilizer) supplied through (N_0_: P_52_: K_34_) after fruit formation (15–20 days interval) up to harvesting of fruits].

### Estimation of tomato yield and fruit quality parameters

The data on plant height/shoot length, growing duration, fruit weight, fruit yield, carotenoid, vitamin C, and lycopene contents were recorded during the growing phase based on five tagged tomato plants in each treatment. The shoot length (m) was measured with measuring tape at the time of final fruit harvest, fruit weight at every harvest, and summed to calculate total yield per plant in tagged plants of tomato. The growing duration was calculated from day one to the last day of fruit harvesting, expressed as days. The 50 g sample of leaves, stem/shoots of tomato was collected from tagged plants and placed in the oven at 60.0 °C for constant weight to calculate dry matter contents in leaves/shoots of tomato plants (kg plant^–1^). The plant materials of tomato variety ‘Himsona’ have been used to estimate the dry matter content under different treatments based on the average of 5 plants each. The tagged tomato plants were uprooted after 90 days when they achieved full maturity. The leaves, stem/shoots were sampled to obtain 50 g samples from each plant. The fruit quality parameters like carotenoid, vitamin C, and lycopene content were analyzed as per the standard procedures^14^. The experiments conducted involving different experimental protocols were approved by Prioritization, Monitoring and Evaluation (PME) cell and Head of the Institution (ICAR-Indian Institute of Soil and Water Conservation), 218 Kaulagarh Road, Dehradun, Uttarakhand. It is stated that all methods have been conducted according to the standard guidelines and regulations. Water use efficiency (WUE) or water productivity (WP) was calculated as the ratio of tomato yield and water applied during the entire growth period from planting to the last day of watering.

### Analysis of nutrient status, microbial population and enzymatic activity of soil

Soil samples were taken with the help of Auger and analyzed in the laboratory. The collected samples were air-dried and ground to pass through a 2 mm sieve. Soil parameters were measured following standard methods viz; soil organic carbon (OC)^15^, total nitrogen^16^, soil pH^17^. The available P was determined color-diametrically by the Olsen method and extracted K by flame photometry_._ Microbial analysis of soil was carried out following standard procedures. The total microbial counts were analyzed by the standard pour plate technique. Soil microbial population count was performed using serial dilution method^17^ and spread on different agar mediums viz. nutrient agar (M001) for bacteria, potato dextrose agar (M096) for fungi, and actinomycetes specific agar (M490) for actinomycetes. The microbial count (population) was expressed as colony-forming units g^−1^ (cfu g^−1^ soil) of dry soil. Soil dehydrogenase activity (DHA) was determined by 24 h of incubation, followed by a pink color intensity measured by spectrophotometer^18,19^. The β-glucosidase activity^20^ and phosphatase assay^21^ were determined by following standard procedures.

### Statistical analysis

The experiment was laid in a randomized block design (RBD) with 3 replications. The data were analyzed using analysis of variance (ANOVA), assuming null hypothesis that all treatments' effect is equal. The post hoc analysis for pairwise treatment analysis was done using Tukey’s Honest Test at p = 0.05 level of significance. The relationship between parameters was analysed using Pearson’s correlation coefficient for all the attributes like shoot length, crop duration, dry matter content in shoots, average fruit weight, fruit yield per plant, water use efficiency, vitamin C, lycopene content, and carotenoid^22^. Further, principal component analysis (PCA) was carried out to eliminate collinearity among growth, fruit production, quality, microbial population, enzymatic activities, and treatment ranking. The hierarchical cluster analysis of treatments using the Euclidean distance method was also performed with principal components (PC) as input instead of all the parameters studied because all variables were highly correlated.

## Results and discussion

### Growth parameters and fruit yield attributes

**Figure 4:**
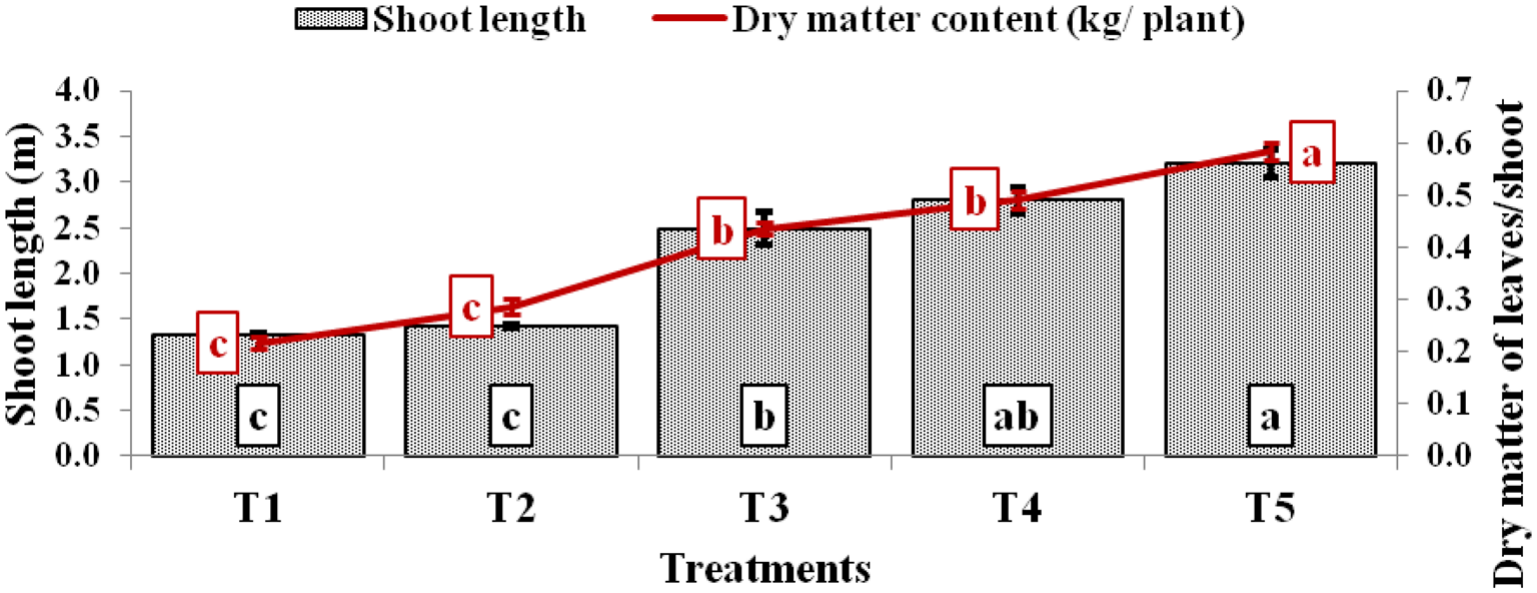
Shoot length (m) and dry matter content of tomato under various treatments.

**Figure 5:**
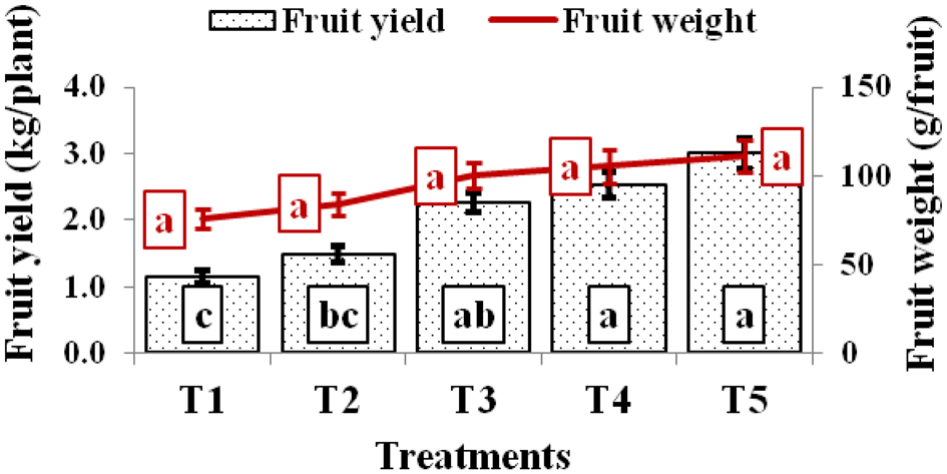
Average fruit weight and fruit yield of tomato under various treatments.

The shoot length, dry matter, fruit weight, and tomato yield varied significantly among the treatments (Figs. 4, 5). The maximum shoot length (3.20 m) and dry matter content (0.584 kg plant^–1^) were observed in tomato plant grown on the raised bed, polythene mulch, regular pruning, trellising, and application of liquid fertilizers (T_5_ treatment) followed by T_4_ (2.67 and 0.491), T_3_ (2.49 and 0.435), T_2_ (1.43 and 0.287) and lowest (1.33 m and 0.217 kg plant^–1^) under T_1_ treatment in case of tomato. The shoot length and the dry matter recorded in T_5_ treatment were 141.21 & 170.5, 124.30 & 103.7, 28.51 & 34.2, and 20.75% & 18.79% higher than T_1_, T_2_ T_3,_ and T_4_ and treatments, respectively (Fig. 4). Similarly, the highest average fruit weight (110.9 g per fruit) was measured with T_5_ treatment followed by T_4_ treatment (105.13 g), T_3_ (100.07 g), T_2_ (83.90 g), and (75.23 g) in T_1_ treatment. The tomato plants raised under T_5_ treatment produced 47.41, 32.18, 10.83, and 5.49% more average fruit weight than T_1_, T_2_ T_3,_ and T_4_ and treatments, respectively (Fig. 5). Likewise, T_5_ treatment also produced the highest fruit yield (3.00 kg plant^–1^ or 102.56 t ha^–1^), which was 160.87, 101.79, 32.74, and 18.89% higher fruit yield over T_1_ (1.15 kg plant^–1^ or 39.32 t ha^–1^), T_2_ (1.49 kg plant^–1^ or 50.83 t ha^–1^), T_3_ (2.26 kg plant^–1^ or 77.26 t ha^–1^) and T_4_ (2.52 kg plant^–1^ or 86.27 t ha^–1^) treatments, respectively (Fig. 5).

Fruit yield is a function of many independent factors like shoot length, fruit weight, number of fruits, nutrient management, pruning, training, and water use. The fruit yield was positively correlated with shoot length (r = 0.91), fruit weight (r = 0.76), dry matter content in leaves (r = 0.91), and water use efficiency (r = 0.96), and the highest fruit yield was recorded under T_5_ treatment. In the integrated approach (T_5_), pruning of the tomato plants removed all food-wasting shoots, thereby improving the growth of the trellised main shoot vertically in the desired direction with regular spraying of liquid fertilizers. This enhanced the vegetative growth, shoot length, dry matter content and partitioned to developing fruit, thereby producing higher fruit yield. However, the lowest productivity in T_1_ treatment may be due to improper plant management, which makes the plant bushier, branchy, compact leading to humid vegetation, poor ventilation making the plants more susceptible to pests/diseases, thereby reducing early growth than advanced treatments (T_3_–T_5_). Previous studies reported similar reasoning of higher tomato yield under pruning, which improved height, fruit yield, light exposure, and better ventilation to each plant and maintained the balance between root and fruit yield over unpruned plant^23–25^. Trellis system of tomato shoot training coupled with pruning improved light penetration, more growing duration, ventilation, photosynthetic efficiency, and 18–25% more fruit yield^26,27^. In another study, higher fruit yield was obtained in tomatoes (106.7 t ha^–1^) with bigger-sized fruits due to a more extended harvesting period in pruned, trained, thinned tomatoes^28^. The dry matter production was positively correlated with fruit yield in tomatoes. In the present study, 12.5–13.0% dry matter had been recorded in tomato shoots. Similarly, other studies found 12.84% dry matter in tomato in open condition^29^ and 9.3–12.5% under polyhouse condition^30^. The fruits are the strongest sinks of assimilates, followed by stem and roots in tomato^31^.

### Water use efficiency (WUE)

**Figure 6:**
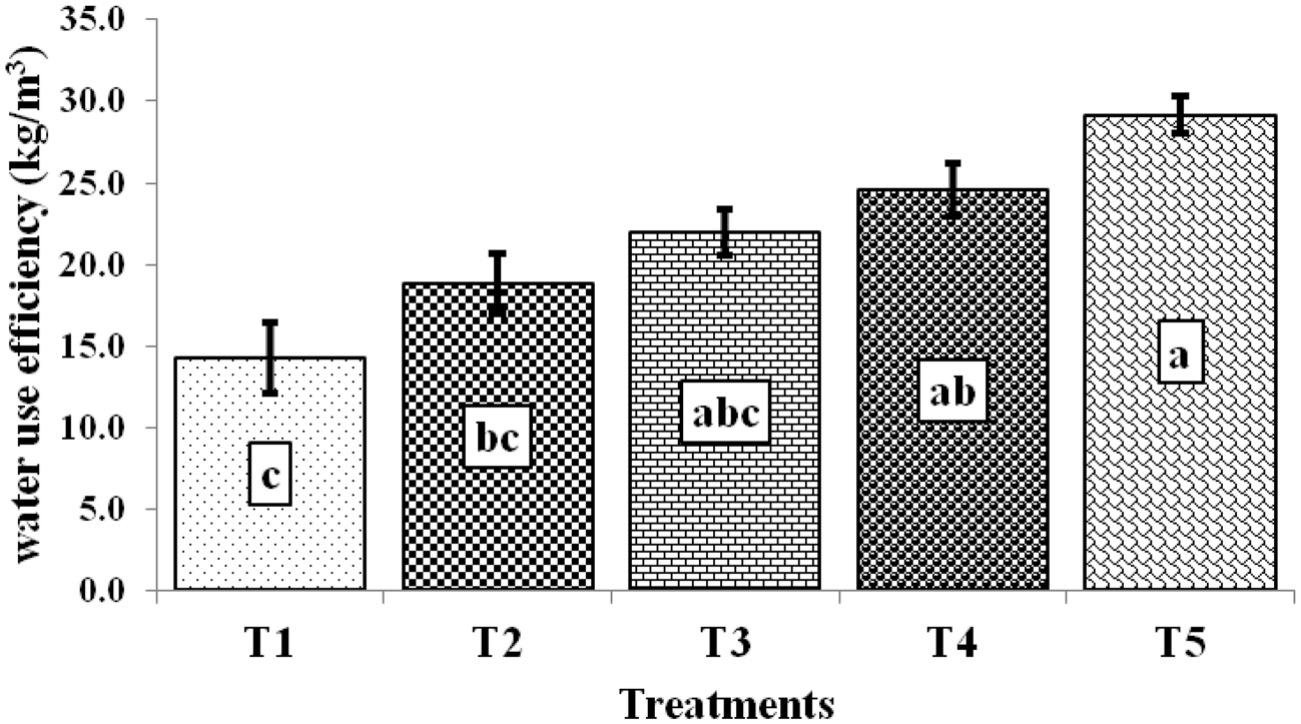
Water use efficiency of tomato under various treatments.

The water use efficiency (kg m^–3^) of tomato differed significantly among treatments (Fig. 6). The maximum WUE of tomato was observed in T_5_ treatment (28.39 kg m^–3^) followed by T_4_ treatment (26.11), T_3_ (22.07), whereas it was minimum (14.30) in T_1_ treatment. The total water applied was 275, 270, and 351.6 mm during the growing period in T_1_, T_2,_ and T_3_–T_5_ treatments, respectively. The 98.60, 39.71, 28.63, and 8.75% higher WUE were observed under T5 treatment compared to T_1_, T_2_ T_3,_ and T_4_ treatments. Similarly, tomato plants grown under T_4_ treatment utilized water efficiently by 82.61, 28.46, and 18.28% more than T_1_, T_2,_ and T_3_ treatments. The water applied was positively correlated with shoot length (r = 0.96) and fruit yield (r = 0.89). It indicated that watering to the tomato plant increased nutrient mobilization within the plant body, which increased shoot length and fruit yield. The previous study had also reported 40% higher water productivity (27.82 kg m^–3^) over control (19.91 kg m^–3^) in tomato^32^. In our study, the higher WUE of tomato in T_5_ treatment (28.39 kg m^–3^) may be due to more photosynthates formation used by developing fruit. Besides, mulching also played a significant role in minimizing evaporation loss over the traditional practice^33^.

### Quality parameters

**Figure 7:**
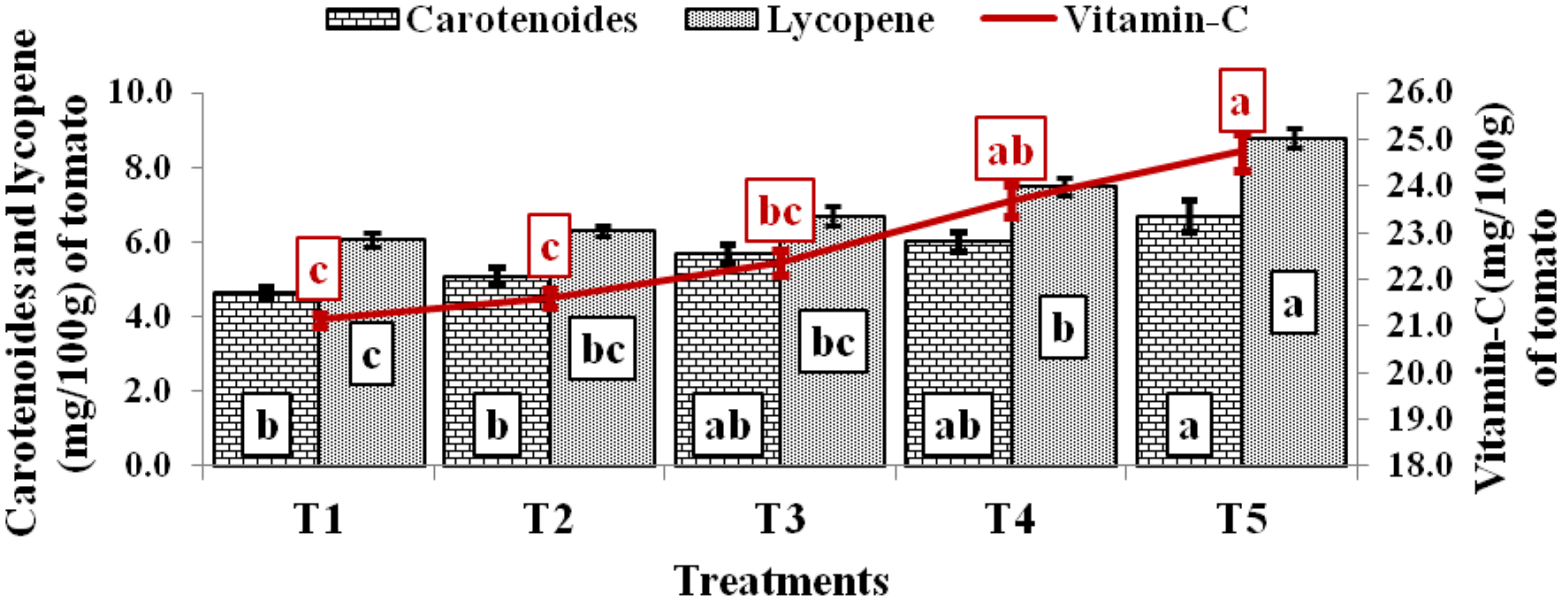
Fruit quality parameters of tomato under various treatments.

The chemical fruit qualities (vitamin C, lycopene, and carotenoid) of tomato varied significantly among treatments imposed on the flatbed and raised bed at farmers' fields (Fig. 7). The highest vitamin C (24.74 mg/100 g) in tomato was found in T_5_, which was 4.40–17.05% higher than T_4_–T_1_ treatments, 5.93–12.11% higher in T_4_ treatment than T_3_–T_1_ treatments, 3.50–5.84% higher observed with T_3_ treatment as compared to T_2_–T_1_ treatment and minimum vitamin C was observed with T_1_ treatment. Similarly, the maximum lycopene (8.78 mg/100 g) of tomato was observed in T_5_ treatment. About 11.49–23.32% higher lycopene was found in T_4_ treatment over T_3_–T_1_ treatments, 6.35–10.62% more lycopene in T_3_ treatment than T_2_ and T1 treatments, and minimum lycopene was observed with T_1_ treatment. Likewise, the highest carotenoid (6.69 mg/100 g) was observed in the T_5_ treatment, 11.51–44.21% higher than T_4_–T_1_ treatments, 5.76–29.33% higher in T_4_ treatment than T_3_–T_1_ treatments, 12.13–22.29% higher carotenoid in T_3_ treatment as compared to T_2_–T_1_ treatments and was minimum with T_1_ treatment. The chemical fruit qualities (vitamin C, lycopene, and carotenoid) of tomato are very important ingredients of the human diet as these act as antioxidants in the human body, which removes free radicals formed during food digestion^34,35^.

Fertilization is an essential element for crop growth and development that plays an indispensable role in accumulating nutrients and aromatic volatiles^36^. Application of higher and additional doses of NPK couple with FYM and training/pruning in tomato improved availability of plant nutrients and growth hormones during the entire phase of growth and development fruit, which elevated photosynthates accumulation utilized by developing fruits and increased fruit yield. The synthesis of vitamin C, lycopene, and carotenoid depends mainly on photosynthates assimilation and supplies the required amount of photosynthates to the developing fruits because fruits are powerful sinks for carbohydrates^37^. The application of FYM improved the availability of organic acid, free amino acids, sugars, and macromolecules that are a direct precursor of synthetic sugar acids; these substances play an essential role in plant metabolism, microbial growth activities, and soil organic matter decomposition^38^. The phosphorus increased ascorbic acid and lycopene in the tomato fruit, even though the moderate application of nitrogen increase tomato yield^39^ and potassium ultimately affects quality by regulating plant carbon and nitrogen metabolism, stomatal opening and closing, enzyme activity, and photosynthetic product transport^40^. The pruning and training in tomato improved fruit qualities significantly because it regulated optimum plant foliage for photosynthesis to meet the carbohydrate requirement of developing fruits without waste. The application of liquid potassium dose @ 4.5 g per liter had significantly improved photosynthesis, enzyme activation, cell turgor maintenance, which had also been observed and reported earlier^41^. The integration of FYM with mulching, training/pruning, and liquid fertilizers improved fruit yield with optimum fruit size and fruit quality parameters.

### Microbial population and enzymatic activities of soil

The microbial population (bacteria, fungi, and actinomycetes) and enzymatic activities (dehydrogenase, β-glucosidase, and phosphatase) of soil varied significantly among treatments (Table 2). The highest microbial population of bacteria, fungi, and actinomycetes were observed as 22.17, 20.67, and 58.67 CFU g^−1^ of dry soil in the case of T_5_ treatment, which was 10.83–111.11, 30.53–226.32, and 7.32–77.78% higher compared to T_4_, T_3_, T_2_, and T_1_ treatments, respectively. Similarly, soil enzymatic activities viz*.* dehydrogenase, β-glucosidase, and phosphatase in the soil were recorded maximum (143, 137.67, and 505.33) with T5 treatment, which registered 8.47–99.07, 11.77–72.30, and 39.72–139.49% higher enzymatic activities in the soil in comparison to T_4_, T_3_, T_2_, and T_1_ treatments, respectively. Organic manure, tomato management practices (training and pruning), and liquid fertilizers increased biomass production, which added higher soil organic matter that increased enzymatic activities. The highest microbial population was recorded in T_5_ treatment because it was supplemented with a maximum dry matter of tomato (19.95 t ha^−1^) in the field coupled with farmyard manure (@ 10.0 t ha^−1^) that provided an adequate amount of food to the microorganisms, which proliferated efficiently in the soil under ambient conditions in the mulched plots. Previous studies^42,43^ also reported higher microbial masses of bacteria, fungi, and actinomycetes in the plots that received farmyard manure integrated with solid fertilizers and green manures. The use of liquid fertilizers integrated with farmyard manure also helped improve the microbial population due to soil conditioning with the organic source. Similarly, enzymatic activities were also recorded highest in the T_5 _treatment due to higher biomass recycling coupled with farmyard manure in the field, which increased soil enzymatic activities. Soil enzyme activities also improved under integrated nutrient management (T_5_ treatment) that received elevated doses of organic manure coupled with training, pruning, and liquid fertilizer application because it provided additional food supply of organic matter under the favourable condition to the microbes compared to farmer practice (T_1_–T_2_ treatments)^44–49^.

**Table 2:** Soil microbial population and enzymatic activities (arithmetic mean ± standard error**)** under different treatments.

Treatment	Bacteria (cfu/g dry soil × 10^6^)	Fungi (cfu/g dry soil × 10^5^)	Actinomycetes (cfu/g dry soil × 10^4^)	DHA (µg TPF released g/24 h of dry soil)	β-Glucosidase (µg PNP released/g/h of dry soil)	Phosphatase activity (µg PNP released/g/h of dry soil)
T1	10.50 ± 1.04^c^	6.33 ± 0.88^c^	33.00 ± 1.15^c^	71.83 ± 1.45^c^	79.90 ± 1.02^c^	211.00 ± 6.43^d^
T2	11.83 ± 1.30^bc^	7.67 ± 0.73^c^	35.33 ± 2.03^c^	75.90 ± 2.89^c^	83.43 ± 2.53^c^	226.17 ± 5.78^d^
T3	16.50 ± 1.44^abc^	10.00 ± 1.15^bc^	45.55 ± 1.13^b^	96.17 ± 3.55^b^	112.60 ± 3.29^b^	313.67 ± 4.91^c^
T4	20.00 ± 2.08^ab^	15.83 ± 1.36^ab^	54.67 ± 1.76^a^	131.83 ± 4.10^a^	123.17 ± 2.33^b^	361.67 ± 8.69^b^
T5	22.17 ± 2.17^a^	20.67 ± 1.20^a^	58.67 ± 2.03^a^	143.00 ± 3.51^a^	137.67 ± 2.64^a^	505.33 ± 5.81^a^

*cfu* Colony forming units, *DHA* Dehydrogenase activity, *PNP* p-nitrophenol, *TPF* Triphenylformazan. The values in same column followed by the same letter are not significantly different according to Tukey’s Honest Test (p = 0.05).

### Profitability

**Figure 8:**
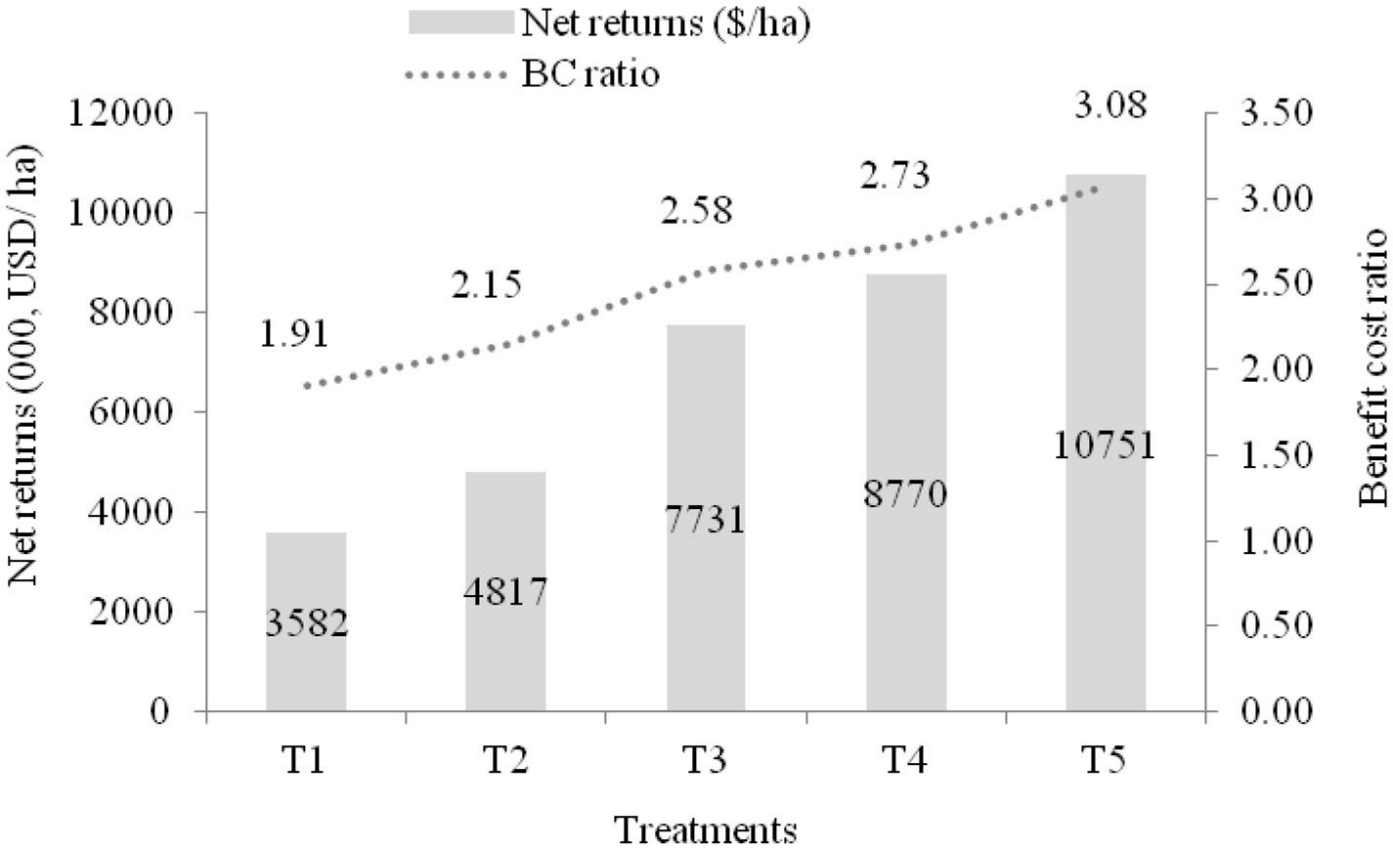
Net returns and BCR of tomato under various treatments.

The net return among various treatments showed an increasing trend from T_1_ to T_5_ treatments. The highest net return (10.75 thousand USD ha^–1^) was recorded with T_5_ treatment followed by T_4_ (8.77), T_3_ (7.73), T_2_ (4.82), and minimum with T_1_ treatment (3.58 thousand USD ha^–1^). The benefit–cost ratio (BCR) of different treatments of pruning, trellising, liquid fertilizer application was calculated and shown in Fig. 8. The highest BCR (3.08) was recorded in T_5_ treatment followed by T_4_ (2.73), T_3_ (2.58), T_2_ (2.15), and minimum with T_1_ treatment (1.91). The BCR indicated that all the treatments were beneficial to the growers as the return was higher than investment, but farmer practice (T_1_ and T_2_ treatments) realized low BCR than improved practices (T_3_, T_4_, and T_5_). From the economic point of view, it is apparent from the above results that the treatment T_5_ was more profitable compared to other treatments. Some authors also observed higher BCR (3.7) under different nutrient levels, pruning and training system of tomato^50,51^. The net return ranged from 7.95 to 11.06 thousand USD obtained from the combination of technologies in tomato compared to traditional farming practices (3.58–4.82 thousand USD). A previous study also showed higher profitability in pruned and staked tomato^10^. The marketable produce harvested was more in advanced treatment (T_3_–T_5_) over farmers’ practice due to balanced nutrient management, aeration in the plant canopy, better photosynthesis by the plant foliage, and its distribution to all developing fruits.

### Principal component analysis and hierarchical clustering analysis

**Figure 9:**
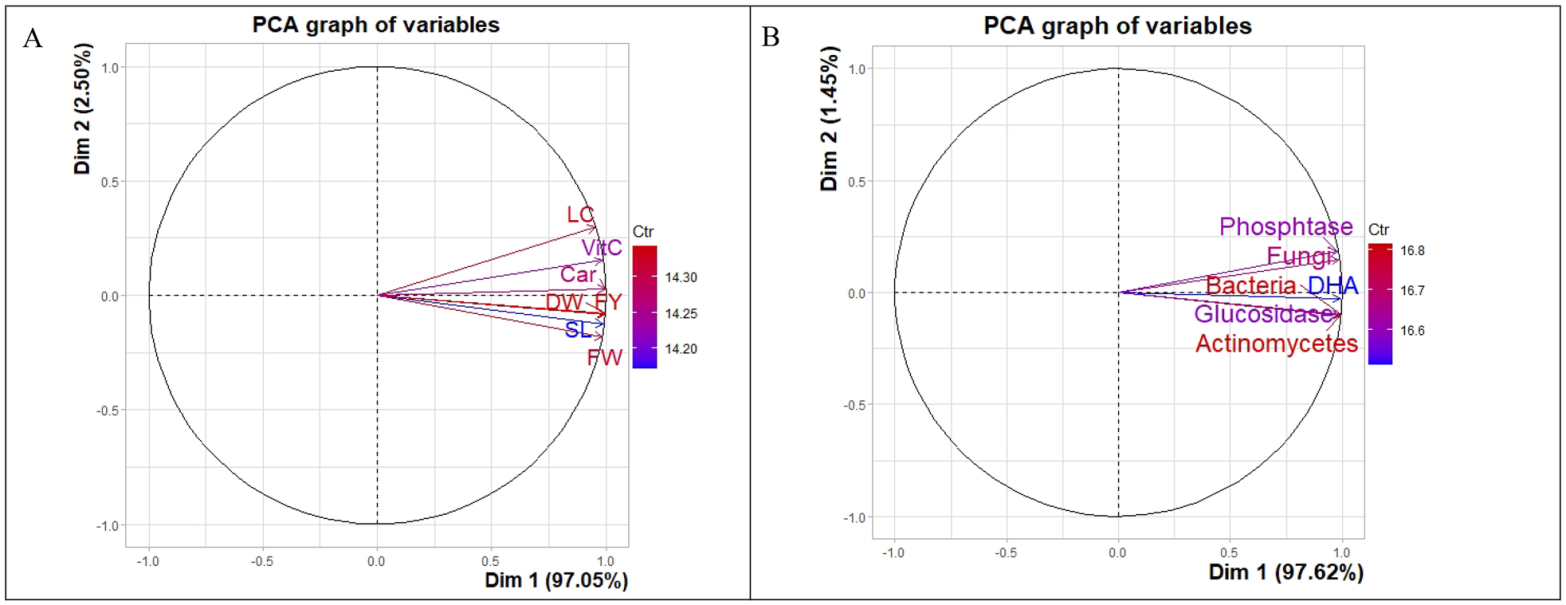
(**A**) Principal component analysis (PCA) represents the contribution of growth parameters/productivity-related parameters on PC1 and PC2. (**B**) Principal component analysis (PCA) represents the microbial population's contribution and enzymatic activities-related parameters on PC1 and PC2.

The principal component analysis was also done as a dimension reduction technique to evaluate the treatment effect on growth/productivity parameters and microbial population / enzymatic soil activities (Table 3A–B). Firstly, principal component analysis (PCA) was done on 7 parameters. The first principal component was selected using the eigenvalue criteria (> 1), which explain 97.05% of the total variance (with communality value ≥ 0.910) of 7 growth/production parameters. In the principal component 1, the highest contribution in terms of factor loading was given by dry matter and carotenoid (0.997), and the lowest contribution yielded by lycopene content (0.954) with performance order dry matter = carotenoid > fruit yield > shoot length > vitamin C > fruit weight > lycopene content. The contributions of the individual growth parameters were expressed in one component model given in Fig. 9A, PC 2 has not contributed significantly (eigenvalue < 1) but had been included in the PCA graph for better interpretation.

**Table 3:** Results of principal component analysis (PCA) showing principal components (PC) with their Eigenvalues and proportion of variance (in percent) explained, along with rotated factor loadings and communalities growth/productivity parameters (A) and for microbial population and enzymatic activities related parameters (B).

A			B		
Parameter	PC1	Communality	Parameter	PC1	Communality
Fruit yield (FY)	0.996	0.992	Bacteria	0.995	0.99
Fruit weight (FW)	0.982	0.964	Fungi	0.985	0.97
Shoot length (SL)	0.986	0.972	Actinomycetes	0.994	0.99
Dry matter (DM)	0.997	0.994	DHA	0.990	0.98
Lycopene content (LC)	0.954	0.910	Glucosidase	0.988	0.98
Vitamin-C	0.984	0.968	Phosphtase	0.976	0.95
Carotenoids	0.997	0.994	Eigenvalue	5.86	
Eigen value	6.793	–	% variance	97.62	–
% variance explained	97.05

Again the principal component analysis (PCA) was conducted on the six selected microbial population/enzymatic activities related parameters (bacteria, fungi, actinomycetes, dehydrogenase, glucosidase, and phosphatase) to screen the treatment effect (T_1_–T_5_) on microbial population/enzymatic activities. The first principal component was selected using the eigenvalue criteria (> 1), which explain 97.62% of the total variance (with communality value ≥ 0.950) of 6 microbial population/enzymatic activities of soil. In the principal component 1, the highest contribution in terms of factor loading was given by bacteria (0.995) and lowest contribution reflected by phosphatase (0.976) with performance order bacteria > actinomycetes > dehydrogenase > glucosidase > fungi > phosphatase. The contributions of the individual parameter (microbial population/enzymatic activities) were expressed in one component model given in Fig. 9B where though cont contributed significantly (eigenvalue < 1) yet shown in the PCA graph for better understanding to know the performance of parameters.

**Figure 10:**
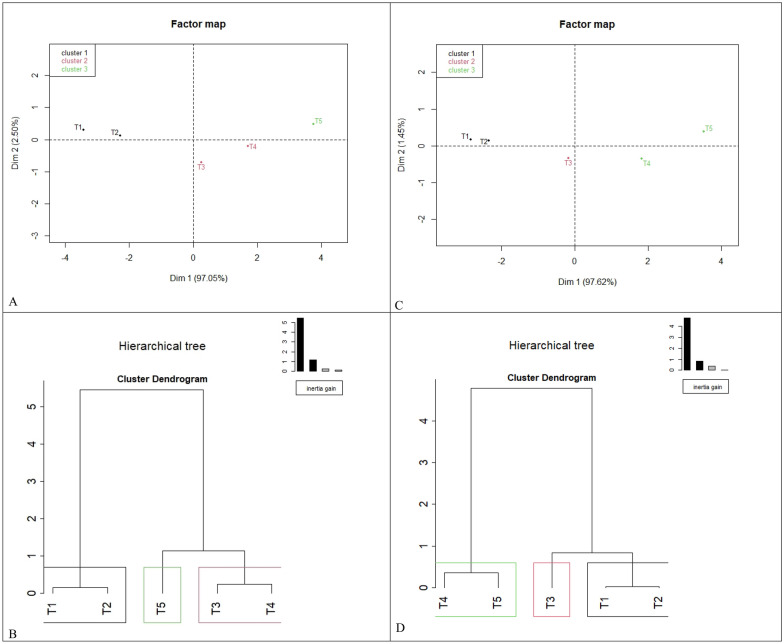
(**A**): Individual factor map of different treatments (T_1_–T_5_) using principal component analysis (PCA) for growth/productivity related parameters, (**B**) Dendrogram obtained by hierarchical cluster analysis (HCA) using PC1 of PCA for growth/productivity related parameters (**C**) Individual factor map of different treatments (T_1_–T_5_) using principal component analysis (PCA) for microbial population and enzymatic activities related parameters, (**D**) Dendrogram obtained by hierarchical cluster analysis (HCA) using PC1 of PCA of microbial population and enzymatic activities related parameters.

The hierarchical clustering analysis (HCA) using PC1 (generated through principal component analysis of 7 growth parameters) was also carried out for grouping of homogeneous treatment and treatment ranking (Fig. 10A,B). The Individual factor map of different treatments (T_1_–T_5_) ranked the treatments as 1st (T5) > 2nd (T4) > 3rd(T3) > 4th (T2) > 5th rank (T1) based on contribution on PC1 (Fig. 10A). The dendrogram obtained from hierarchical cluster analysis of PC1 (Fig. 10B) where cluster 1 with lowest treatment effects contains (T_1_ and T_2_), Cluster 2 with moderate treatment effects contains (T_3_ and T_4_), and cluster 3 with highest treatment effects contains only (T_5_). Similarly, the hierarchical clustering analysis (HCA) using PC1 (generated through principal component analysis of 6 soil-related parameters) was also carried out for grouping of homogeneous treatment and treatment ranking (Fig. 10C,D). The Individual factor map of different treatments (T_1_–T_5_) ranked shown a similar pattern already reported in growth/productivity parameters (Fig. 10C). The dendrogram structure of hierarchical cluster analysis obtained was also more or less similar to growth/productivity parameters except for T_4_ treatment, which shifted from cluster 2 (moderate) to cluster 3 (highest) of PC1 (Fig. 10D).

## Conclusion

This study on tomato was conducted to improve productivity, quality, and net returns through plant architectural modification such as trellising and pruning and liquid fertilizer use. The integrated approach, i.e. T_5_ (trellising, pruning, and liquid fertilizers in tomato grown on a raised bed with polythene mulch) improved fruit yield, shoot length, dry matter content, water productivity, vitamin C, carotenoid, lycopene with a net return of 10.75 thousand USD ha^–1^ in comparison to farmer practice. Therefore, an integrated approach is beneficial to the farmers for higher production with quality fruits and net returns. However, it is imperative to gain knowledge and expertise in trellising and pruning with liquid fertilizers to achieve a higher fruit yield with a better tomato quality. This integration approach of trellising, pruning and liquid fertilizers will improve farmers' economic status and reduce pests and diseases in the field by providing well-placed plants, branches, and fruits. The clustering and ranking of all the parameters and treatments also confirmed the superiority of the integrated approach over other treatments in terms of tomato fruit production and quality.
